# Lymph Node Sampling Patterns and Completeness of Staging During Systematic Mediastinal Lymph Node Staging in Patients with Locally Advanced Non-Small-Cell Lung Cancer: A Post Hoc Analysis from the SEISMIC Study

**DOI:** 10.3390/cancers18111766

**Published:** 2026-05-28

**Authors:** Kanishka Rangamuwa, Ashleigh Witt, Joseph M. Curran, Gargi Kothari, Neil Wallace, Nicholas Hardcastle, Shaun Yo, Farzad Bashirdazeh, Phan Nguyen, Barton R. Jennings, David Fielding, Laurence Crombag, Kazuhiro Yasufuku, Jouke T. Annema, David Ost, Shankar Siva, Daniel P. Steinfort

**Affiliations:** 1 Department of Respiratory and Sleep Medicine, Royal Melbourne Hospital, 300 Grattan Street, Melbourne, VIC 3050, Australia; kanishka.rangamuwa@mh.org.au (K.R.); ashleigh.witt@mh.org.au (A.W.); josephmcurran@gmail.com (J.M.C.); 2 Faculty of Medicine, Dentistry & Health Sciences, University of Melbourne, Parkville, VIC 3010, Australia; 3 Division of Radiation Oncology, Peter MacCallum Cancer Centre, Melbourne, VIC 3000, Australia; 4 The Sir Peter MacCallum Department of Oncology, University of Melbourne, Parkville, VIC 3010, Australia; 5 Centre for Medical Radiation Physics, University of Wollongong, Wollongong, NSW 2500, Australia; 6 Department of Lung and Sleep, Monash Health, Melbourne, VIC 3168, Australia; 7 Department of Thoracic Medicine, Royal Brisbane and Women’s Hospital, Brisbane, QLD 4006, Australia; 8 Department of Thoracic Medicine, Royal Adelaide Hospital, Adelaide, SA 5000, Australia; 9 Department of Pulmonology, Amsterdam University Medical Centres, University of Amsterdam, 1012 WP Amsterdam, The Netherlands; 10 Division of Thoracic Surgery, Toronto General Hospital, Toronto, ON M5G 2C4, Canada; 11 Department of Pulmonary Medicine, MD Anderson Cancer Center, The University of Texas, Houston, TX 78712, USA

**Keywords:** bronchoscopy, synoptic reporting, staging

## Abstract

**Introduction:** Systematic endoscopic mediastinal lymph node (LN) staging in early stage non-small-cell lung cancer (NSCLC) is widely recommended for optimal pre-operative staging due to imperfect accuracy of PET/CT. The SEISMIC study utilized synoptic reporting of findings during systematic endoscopic staging to inform radiotherapy planning in patients with locally advanced NSCLC and established a significant rate of discordance between PET-identified extent of disease and observed findings following EBUS. International guidelines recommend systematic LN staging in patients with clinical nodal stage (cN) 1–3 disease as a quality indicator, though performance metrics for quality assessment of systematic endoscopic mediastinal staging in NSCLC have not been described. **Methods:** A post hoc analysis of 155 participants with locally advanced NSCLC recruited to the SEISMIC study who underwent systematic endoscopic mediastinal staging, with sampling of all LN ≥ 6 mm, prior to planning of radical or high-dose palliative radiation, was completed. Synoptic procedural reports and pre-procedure PET imaging were examined to determine the number of LN sampled, as well as the number of radiologically normal LN (<10 mm diameter, no PET-avidity) sampled, and to establish the completeness of mediastinal staging. **Results**: Sampling was performed from a median two LN per patient. Sampling from at least one radiologically normal LN was performed in 92/155 (59%) participants, with ≥2 radiologically normal LN sampled in 47 participants (30%). PET-occult LN disease was identified in 18 of 92 (20%) of patients who underwent sampling of radiologically normal LN. The number of LN sampled in individual patients was largely influenced by the number of observed LN ≥ 6 mm in the short-axis dimension. Among these 76 participants staged cN2a by PET, EBUS identified PET-occult metastases in seven (9%), with four (5%) upstaged to cN2b and three (4%) to cN3. In only three patients (2% of cohort) were LN > 6 mm at higher station than the highest LN identified by PET not sampled endoscopically, confirming a high rate of complete mediastinal assessment. **Conclusions**: Quality of systematic staging in the SEISMIC study was high. Synoptic reporting supports high quality systematic endoscopic mediastinal LN staging in NSCLC and enables performance monitoring of systematic EBUS staging. Novel quality metrics are proposed for quality assessment, and consistent use of these may encourage more extensive LN sampling.

## Simple Summary

The SEISMIC study demonstrated the importance of systematic mediastinal LN staging in patients with locally advanced NSCLC undergoing curative-intent radiation, with detection of PET-occult lymph node (LN) metastases having major implications for accurate radiation field planning. This post hoc analysis of procedural outcomes used synoptic procedural reports to evaluate the quality of EBUS sampling performed and to define potential, measurable quality indicators in systematic endoscopic staging procedures. Among the SEISMIC cohort, synoptic reports enabled assessment of procedural quality measures, including confirming sampling of radiologically ‘normal’ lymph nodes in a high proportion of patients and a very high rate of adherence to recommended systematic LN sampling practice. The number of LN sampled in individual patients was largely influenced by the number of observed LN ≥ 6 mm in the short-axis dimension. This analysis confirms systematic endoscopic staging as a critical component of accurate disease assessment and proposes a framework for improving quality and consistency in mediastinal staging.

## 1. Introduction

Consistent use of Positron Emission Tomography (PET) for pre-operative staging to confirm early stage non-small-cell lung cancer (NSCLC) reduces rates of post-operative pathologic upstaging; however, PET-occult disease is still observed in approximately 6–9% of patients, with rates of pathologic up-staging varying according to clinico-radiologic factors, including a tumor size >3 cm and clinical node (cN) stage [1,2,3]. Accordingly, systematic mediastinal lymph node (LN) staging for NSCLC in selected pre-operative candidates has been recommended in international consensus guidelines since 2015 [3,4,5].

Systematic staging describes stepwise evaluation of all mediastinal LN stations, with sampling via transbronchial needle aspiration (TBNA) of any LN ≥ 6 mm in size [6]. Significant variation in number of LN sampled during systematic mediastinal staging with endobronchial ultrasound (EBUS) is recognized, ref. [7], with studies reporting a higher mean number of LN sampled frequently demonstrating greater sensitivity for detection of PET-occult mLN metastases [8,9]. Less extensive LN sampling during staging EBUS may have significant consequences for procedural outcomes—there is a linear relationship between the number of lymph nodes examined and the odds of upstaging (odds ratio as high as 6.1) following surgical resection [10]. Equally, the impact on clinical outcomes in resected NSCLC is illustrated by the prognostic utility of the International Association for the Study of Lung Cancer (IASLC)—Lung Cancer Staging Project—Residual Tumor Descriptors, specifically in evaluating survival of patients with uncertain residual tumor status (R*(un)*) following inadequate intra-operative LN staging [11].

The SEISMIC study was a prospective multicenter single-arm clinical trial [12] that applied systematic endoscopic mediastinal lymph node staging before combination chemoradiotherapy or high-dose palliative radiotherapy in patients with suspected or known locally advanced NSCLC. Endoscopic sampling was performed utilizing synoptic reporting, where findings were recorded at all mediastinal LN stations. PET-occult disease was identified in twelve percent of patients, with in silico radiation planning studies confirming that patients for whom systematic endoscopic mediastinal staging demonstrates PET-occult disease would receive a suboptimal radiotherapy dose if planned based upon PET-avid disease alone, contributing to a significantly higher likelihood of disease relapse following curative intent chemoradiotherapy. Additionally, twenty-five percent of patients were found to have a lesser extent of disease than observed on PET imaging [9].

This post hoc analysis examines lymph node sampling patterns in participants with locally advanced NSCLC undergoing systematic endoscopic staging as part of the SEISMIC study. Recognizing that the extent of LN sampling performed may have an impact on staging accuracy (including rate of detection of PET-occult LN metastases), we aimed to establish the completeness of LN sampling performed in the SEISMIC study and to identify any potential for improved sampling patterns that may increase the detection rate of PET-occult disease in this patient group.

## 2. Methods

The study protocol and primary manuscript have both been previously published [12,13]. Briefly, this prospective single-arm study recruited 155 participants with locally advanced NSCLC across seven sites in four countries. Patients aged 18 years and older with known or suspected locally advanced or unresectable NSCLC, based on CT and PET findings, were included if they were considered potential candidates for radical dose or high-dose palliative conventional radiotherapy (with or without chemotherapy). Patients underwent EBUS-TBNA, either as their primary diagnostic procedure or as a dedicated staging procedure following a previous diagnosis of NSCLC, with systematic examination of mediastinal lymph node stations commencing at the highest echelon mediastinal lymph node visualized (i.e., N3) and proceeding more proximally. Sampling was performed from any visible lymph node 6 mm or larger.

Both PET/CT findings and EBUS imaging findings were used to record findings at all five mediastinal LN stations (2R, 2L, 4R, 4L, and 7). PET/CT characteristics recorded at each LN station included short-axis dimension (mm) and FDG-avidity (Y/N). Synoptic procedural reporting was used to ensure findings at all LN stations were recorded as follows:

- LN station
- Lymph node seen Y/N
- (if yes) LN size (short axis, in mm)
- (if ≥5 mm) LN sampled Y/N

Pathologic outcomes for all sampled LN stations were also recorded.

Performance of combined endoscopic ultrasound using the EBUS videobronchoscope (EUS-B) [14] was at proceduralist discretion. Where this was performed, findings and procedural sampling were performed similar to above, with synoptic reporting requiring recording of findings at stations 4L, 5, 7, and 8.

In addition to completeness of endoscopic mediastinal evaluation, we aimed to assess quality of mediastinal staging by determining the proportion of patients in whom radiologically normal LN (≤10 mm, non-FDG-avid) were sampled.

### Statistical Analysis

Given the exploratory nature of this post hoc analysis, results are presented using descriptive statistics only. Continuous variables were assessed for normality using the Shapiro–Wilk test; normally distributed data are expressed as means with standard deviations (SD), while non-normally distributed data are reported as medians with interquartile ranges (IQR). Categorical variables are presented as absolute frequencies and percentages (%). No formal comparative statistics or *p*-values were calculated.

## 3. Results

A total of 155 patients with locally advanced NSCLC underwent systematic endoscopic mediastinal lymph node staging during the study. Discrepancy in extent of mediastinal disease identified by PET and EBUS-TBNA was observed in 57 (37% (95% CI 29–44)) patients. PET-occult lymph node metastases were identified in 18 (12% (7–17)) participants, including 16 (13% (7–19)) of 123 participants with clinical stage IIIA or cN2 NSCLC. Contralateral PET-occult N3 disease was identified in nine (7% (2–12)) of 128 participants staged cN0, cN1, or cN2.

### 3.1. Completeness of Endoscopic Sampling

Details regarding the extent of endoscopic (EBUS + EUS-B) sampling are presented in Table 1. Dedicated findings from EUS-B-FNA are presented in Table 2. Endoscopic sampling was performed from a median 2 LN stations per participant (IQR 1–3). Among 128 patients with cN0/1/2 stage on PET, sampling from contralateral station 4 (N3) was performed in 58 (45%) patients, 21 (36%) with left-sided tumors and 37 (64%) with right-sided tumors. Detection of PET-occult N3 LN metastases was observed in nine participants, comprising 16% of all patients who underwent N3 sampling.

Radiologically normal lymph nodes (i.e., <10 mm short axis on CT, and no FDG-avidity on PET) were sampled in 92/155 (59%) patients. Where no radiologically normal LN were sampled, in 55/63 (87%), this was a result of no LN being observed on EBUS at higher echelon stations above PET-positive LN. In the remaining eight patients (5% of cohort), radiologically normal LN unsampled were at lower echelon stations than a confirmed EBUS-positive LN site in five. Therefore, only three patients (2% of cohort) who had a higher station LN identified on EBUS inspection were left unsampled. Notably, two of the unsampled LN were at the contralateral station 4 (N3).

### 3.2. Sampling of Radiologically Normal LN

Eighty-nine patients had radiologically normal LN sampled, with median size of normal LN sampled 7 mm (IQR 5.7–8 mm), with all of these being from a higher echelon than the highest observed abnormal LN on PET/CT. Sampling of contralateral station 4 LN in patients staged cN0-2 (*n* = 128) was performed in 77 patients with 56 of these being <10 mm.

Ninety-five LN sampled were ≤7 mm, with PET-occult LN metastases identified in five of these (5%, 95%CI 2–12%). Thirty-nine LN sampled were ≤5 mm, with PET-occult metastases identified in one of these LN (3%, 95%CI 0.1–14).

### 3.3. EUS-B Sampling

Thirty-two patients (21%) underwent EUS-B-FNA. PET-occult disease was demonstrated in 1/32 (3%), notably in a 4L LN not able to be sampled by EBUS-TBNA.

### 3.4. PET-Occult LN Metastases

PET-occult LN metastases were identified in 22 LN from 18 patients (20% of 89 patients in whom radiologically normal LN were sampled). Station 4R/L was the commonest location (n = 5/9), with nine (6% of cohort) of these resulting in upstaging to N3 status. A total of 112 patients were staged as cN2 following PET, including 76 as cN2a. Among these 76 participants staged cN2a by PET; EBUS identified PET-occult disease in seven (9%), with upstaging to cN2b observed in four (5%) and to cN3 in three of 76 (4%). Importantly, LN sampling at higher echelon LN than the highest pathologic LN seen on PET was performed in 60 of 76 participants staged cN2a following PET.

Patients with PET-occult disease observed at station 2 (2L = 1, 2R = 3) all had PET-positive ipsilateral station 4 LN. All four patients with PET-occult metastases detected at station 7 had right-sided tumors. Two of these patients had cN1 disease on PET, with station 7 being the sole site of PET-occult metastases.

## 4. Discussion

The SEISMIC study is a landmark study in the management of patients with locally advanced NSCLC which confirmed a significant rate of discordance between PET-identified extent of disease and that established following systematic mediastinal staging with EBUS [12]. PET-occult LN metastases were observed in 12% of patients. In silico radiation studies confirmed a high likelihood of treatment failure at sites of PET-occult disease following definitive chemoradiotherapy, emphasizing the importance of systematic endoscopic mediastinal LN staging in patients with locally advanced NSCLC.

This post hoc analysis utilized synoptic reports completed as part of the SEISMIC study to confirm a high rate of complete mediastinal staging, as demonstrated by a proportion of patients in whom radiologically normal LN were sampled, and a very low rate (2%) of patients where EBUS-visualized ‘normal’ LN at higher echelon stations than PET-detected LN were unsampled. Where patients did not undergo sampling of radiologically normal LN, this appeared to be in almost all cases due to absence of visualized LN at individual LN stations.

These findings that indicate the generalizability of the findings of the SEISMIC study are likely to be highly contingent on high-quality endoscopic staging, emphasizing the importance of process measures to improve consistency and quality of systematic EBUS staging in NSCLC. A high variance in care is consistently noted regarding the frequency of systematic endoscopic LN staging [15,16,17] despite this being a key quality indicator in guidelines describing quality indicators for performance monitoring in EBUS [6].

Although systematic mediastinal staging requires that all EBUS-accessible mediastinal LN stations be examined, the extent of LN sampling may vary considerably, depending on intra-procedural findings and on local practice. Our findings suggest that observed variance in sampling may partly reflect intra-procedural findings; however, in the absence of comprehensive reporting (as enabled by synoptic reports), it is not possible to confirm this suggestion in previously published studies. Notably, performance metrics for quality assessment of systematic endoscopic mediastinal staging in NSCLC have not been described. Our analysis supports the use of metrics, including in a proportion of patients in whom radiologically normal LN are sampled as a process measure, [18], in addition to the clinically impactful outcome measure of rate of detection of PET-occult LN metastases. Assessment of these metrics is greatly aided by synoptic reporting [19], which permits reporting of outcomes such as proportion of patients with radiologically normal LN sampled.

Recent studies have suggested that inadequate and incomplete procedural reporting compromises the assessment of quality in EBUS, [20], and in this edition of the journal, a pilot study confirmed implementation of synoptic procedural reporting improved quality of process measures (including mean number of LN sampled, and proportion of patients with at least one radiologically normal LN sampled), as well as increasing the rate of detection of PET-occult nodal metastases from 3.1% to 11.7% (*p* = 0.036) [21].

The size of LN in which PET-occult metastases were detected in the SEISMIC trial was small, confirming comprehensive sampling is required for optimal accuracy of staging. In patients where sampling of normal LN (sized 5–10 mm) was performed, the prevalence of mediastinal LN metastases was 20%, including 16% of contralateral station 4 (N3) LN. Therefore, understanding the proportion of patients where absence of sampling reflects a missed opportunity to confirm non-invasive cN-staging findings remains a potential quality improvement step.

The clinical impact of failure to sample more proximal/superior LN may be illustrated by the work of the IASLC Lung Cancer Staging Project in describing R-descriptors for resected NSCLC, specifically in evaluating clinical outcomes for patients with uncertain residual tumor status (R*(un)*) [11]. This followed recognition of the impact of method of N-staging (clinical versus pathological) on survival in resected NSCLC—data collected for the 8th edition of the TNM classification demonstrated a difference of as much as 18% in 5-year survival for resected T3 NSCLC according to whether cN0 or pN0 [22].

Uncertain residual tumor status was recognized by the IASLC where the highest mediastinal LN resected was positive, or where incomplete intra-operative mediastinal LN sampling was performed. There was a difference in median survival of 20 months between pN-positive R0 and R*(un)* cases (70 months versus 50 months) (HR = 1.27, *p* < 0.001) [11]. These findings have been externally validated including among 37,149 patients confirming inferior 5-year overall survival (OS) (RR 1.31) for R*(un)* versus R0 patients [23].

Extrapolating from these observations, patients where observed ‘normal’ LN remain endoscopically unsampled (akin to endoscopic R*(un)*) prior to chemoradiation may be at risk of poorer outcomes, as observed in surgically treated patients with R*(un)* status, though evidence regarding this is lacking. Similarly, patients with sampling of radiologically normal LN may be considered to have ‘endoscopic R0’ disease. This information may only be obtained from detailed and standardized procedural reports, such as utilized in the SEIMSIC trial.

Evidence supporting the impact of EBUS/endoscopic staging on mortality is scant, though a recent study of over 1089 patients undergoing EBUS-TBNA suggested endoscopic nodal staging outcomes (eN) held some prognostic value, with survival in patients with eN0–1/pN2–3 being not significantly different than in patients with eN0–1/pN1 and pN1 and 5-year overall survival 50% higher among patients with eN2a vs. eN2b [24]. This is supported by observational findings indicating no impact on rates of unforeseen N2 disease or survival in NSCLC despite endoscopic staging replacing mediastinal staging [25]. Similarly, the MEDIASTrial—a multicenter non-inferiority trial randomly assigning patients after negative endosonography to immediate lung tumor resection or to mediastinoscopy first [26]—reported equal two-year overall and disease-free survival between the randomization groups, [27], despite the immediate resection arm experiencing higher rates of resected unforeseen N2 (uN2) at definite surgical resection. No survival data are available on survival according to eN-stage in patients with locally advanced NSCLC; however based on findings of in silico analysis within SEISMIC, eN-stage may more accurately predict survival than PET-determined N-stage, though this remains to be confirmed in future studies.

A systematic review examining performance of pre-operative systematic staging using EBUS in NSCLC noted variable extent of lymph node sampling performed, with a majority of studies reporting a mean number of LN sampled being less than two [7]. An updated meta-analysis evaluating systematic endoscopic staging including patients with cN1/2 NSCLC confirmed significant variation in number of LN sampled, though few studies reported the number of radiologically normal LN sampled, [28], making evaluation of the quality of endoscopic systematic staging difficult to discern.

One potential aspect of systematic staging identified where completeness of sampling may be further improved is through more consistent and more complete performance of EUS-B-FNA, in jurisdictions where this is feasible. This was undertaken in only 34% of participants, with only 21% undergoing EUS-B-FNA. EUS-B-FNA can be performed safely and accurately within the same anesthetic by pulmonologists [29,30]. Prior meta-analysis confirms improved sensitivity when EUS-B is added to EBUS, particularly where LN are not within reach (e.g., small 4L LN) of EBUS-TBNA [31]. Studies have suggested EUS-B-FNA may halve the ‘Number Needed to Treat’ [7] and should ideally be incorporated where possible into standard systematic staging protocols [29,30].

The SEISMIC study was a prospective registered clinical trial (ACTRN12617000333314); however findings reported above represent a post hoc analysis of trial data and therefore should be interpreted as hypothesis-generating rather than confirmatory. Performance of EUS-B during the trial was ad hoc, and therefore the potential contribution of this technique to accuracy of mediastinal sampling cannot be inferred, though previous studies have indicated the possible impact of performing EBUS-B in addition to EBUS LN staging in NSCLC [7,31]. The impact of synoptic reporting on quality in EBUS staging, as well as the utility and feasibility of proposed performance metrics, require validation in future prospective studies.

## 5. Conclusions

Evaluation of synoptic procedural reports confirms a high rate of completeness of mediastinal staging in the SEISMIC study. A median size of LN with PET-occult metastases detected indicates that the current recommendation of sampling of all visualized LN ≥6 mm remains optimal practice. Synoptic reporting was essential in determining the proportion of patients in whom radiologically normal LN were sampled, and the number of patients in whom systematic mediastinal sampling prior to chemoradiation was incomplete (endoscopic R*(un)*). Further studies are required to confirm the utility of these two metrics as novel process measures for performance monitoring in systematic endoscopic staging in NSCLC.

## Figures and Tables

**Table 1:** Endoscopic sampling patterns.

Number Mediastinal LN Stations Sampled		Radiologically Normal LN Sampled		Median Size ‘Normal’ LN Sampled (mm, Range)	Mediastinal LN ≥ 6 mm Diameter Not Sampled	PET-Occult Mediastinal LN Demonstrated
LN (n)	participants (n)	LN (n)	participants (n)			
5	1	3	1	9 (8–10)	0	0
4	7	4 ^&^	2	6 (4–8)	0	0
		3	2	7 (6–8)	0	0
		2	2	7 (6–8)	0	1
		1	1	4	0	0
3	57	3 ^&^	4	8 (3–10)	0	2
		2	34	7 (4–10)	0	4
		1	14	8 (5–10)	0	2
		0	5	N/A	2 *	0
2	49	2 ^&^	3	8 (4–10)	0	2
		1	25	7 (5–10)	1 *	5
		0	21	N/A	0	0
1	41	1	2	7 (5–9)	0	1
		0	39	N/A	5 *	0

^&^ all patients staged cN0/1 by PET/CT. * one patient in each of these groups staged R(uncertain) due to failure to sample a radiologically normal LN observed on EBUS to be >6mm short axis dimension. N/A, not applicable as no LN sampled.

**Table 2:** EUS-B sampling.

Number LN Stations Sampled		Number EUS-B-FNA from LN Not Sampled by EBUS	Number Patients Radiologically LN Stations Sampled	PET-Occult LN Demonstrated	EUS-B-FNA Sole Method of Demonstrating LN Metastases **
2	8	4 *	5	4	1
1	24	5	10	0	1

* includes two patients where sampling was from sites inaccessible to EBUS-TBNA. ** PET-occult disease was identified at one 9 mm PET-negative 4L lymph node unable to be sampled by EBUS-TBNA, resulting in upstaging from N2 to N3. One 12 mm PET-positive subcarinal lymph node confirmed cytologically by EUS-B-FNA following benign findings at EBUS-TBNA resulted in concordance with PET stage.

## Data Availability

The data presented in this study are available on request from the corresponding author.
